# Current Evidence and Future Perspectives on Pharmacological Treatment of Calcific Aortic Valve Stenosis

**DOI:** 10.3390/ijms21218263

**Published:** 2020-11-04

**Authors:** Maristella Donato, Nicola Ferri, Maria Giovanna Lupo, Elisabetta Faggin, Marcello Rattazzi

**Affiliations:** 1 Department of Pharmaceutical and Pharmacological Sciences, University of Padova, 35122 Padova, Italy; maristella.donato@studenti.unipd.it (M.D.); nicola.ferri@unipd.it (N.F.); mariagiovanna.lupo@studenti.unipd.it (M.G.L.); 2 Department of Medicine—DIMED, University of Padova, 35122 Padova, Italy; elisabetta.faggin@unipd.it

**Keywords:** aortic valve, stenosis, calcification, valvular interstitial cells, calcific aortic valve disease

## Abstract

Calcific aortic valve stenosis (CAVS), the most common heart valve disease, is characterized by the slow progressive fibro-calcific remodeling of the valve leaflets, leading to progressive obstruction to the blood flow. CAVS is an increasing health care burden and the development of an effective medical treatment is a major medical need. To date, no effective pharmacological therapies have proven to halt or delay its progression to the severe symptomatic stage and aortic valve replacement represents the only available option to improve clinical outcomes and to increase survival. In the present report, the current knowledge and latest advances in the medical management of patients with CAVS are summarized, placing emphasis on lipid-lowering agents, vasoactive drugs, and anti-calcific treatments. In addition, novel potential therapeutic targets recently identified and currently under investigation are reported.

## 1. Introduction

Calcific aortic valve stenosis (CAVS) is the most common heart valve disease in the Western World and the third cause of cardiovascular disease after coronary artery disease and systemic arterial hypertension [1]. The prevalence increases with advancing age, reaching 12% in the elderly (>75 years) [2,3]. Its prevalence and impact on public health are expected to increase due to higher life expectancy and the rapid aging of populations worldwide [4]. The disease remains asymptomatic until it reaches the severe stage, where it manifests with syncope, angina, and heart failure, and requires the substitution of the valve [5].

To date, there are no effective medical therapies to halt or delay CAVS progression and the only available option to treat subjects with symptomatic severe CAVS remains surgical or transcatheter aortic valve replacement (AVR) with a mechanical or bioprosthetic valve. However, the two techniques are associated with significant complications: the implantation of a mechanical valve increases the risk of thrombosis and requires a life-long anticoagulation therapy. On the other hand, the bioprosthetic valves are subject to deterioration, which limits their durability and can lead to reoperation in less than 15 years [6]. The development of an effective medical treatment is a major medical need in order to reverse the progression of CAVS, to improve clinical outcomes, and to reduce the need for AVR.

## 2. Methods

The PubMed, Scopus, and Google Scholar databases were searched by using the key terms “calcific aortic valve disease”, “aortic valve stenosis”, and “valvular interstitial cells”, selecting the most relevant articles. The clinical trials were searched in the clinicaltrial.gov database by selecting “aortic valve stenosis” as the disease. In addition, the proposed treatments were searched in databases associated with the key terms “aortic stenosis”, “vascular calcification”, and “atherosclerosis”. Reference lists from these papers were then searched to identify previous studies. This review focuses on patients with a tricuspid aortic valve, except when otherwise specified.

## 3. Pathophysiology of CAVS

CAVS is the slowly progressive fibro-calcific remodeling of the valve leaflets, resulting in reduced mobility, gradual narrowing of the valve, and progressive obstruction to the blood flow. Traditionally, it was considered an age-related degenerative process caused by passive calcium deposition in the valve. Nowadays, its pathophysiology has been further investigated and its disease progression has been related to active processes involving cellular and molecular pathways.

CAVS is a multi-step disease that can be divided in two distinct phases: an early initiation phase and a later propagation phase, each characterized by different mechanisms. The initiation phase, termed aortic sclerosis, shows similarities with atherosclerosis and both conditions share common risk factors (age, male gender, smoking, hypertension, dyslipidemia, metabolic syndrome) [7]. The initiating event is represented by an endothelial damage on the aortic side of the valve, due to increased mechanical stress and reduced shear stress. The loss of endothelial integrity facilitates the infiltration of monocytes, mast cells, T cells, and lipoproteins (such as low density lipoprotein (LDL), lipoprotein(a)), promoting inflammation and lipid accumulation [8]. Once in the subendothelium, monocytes are activated to macrophages, T cells release pro-inflammatory cytokines (interleukin (IL)-1, IL-6, tumor necrosis factor (TNF)-α) and LDL undergo oxidation to oxidized LDL (oxLDL), which are recognized by macrophage scavenger receptors and give rise to foam cells. These processes induce further oxidative stress and inflammatory response. Although inflammation and lipid deposition may be important in establishing the disease, their role becomes less prominent in the propagation phase, which is mainly characterized by fibrosis and calcification [9].

**Figure 1:**
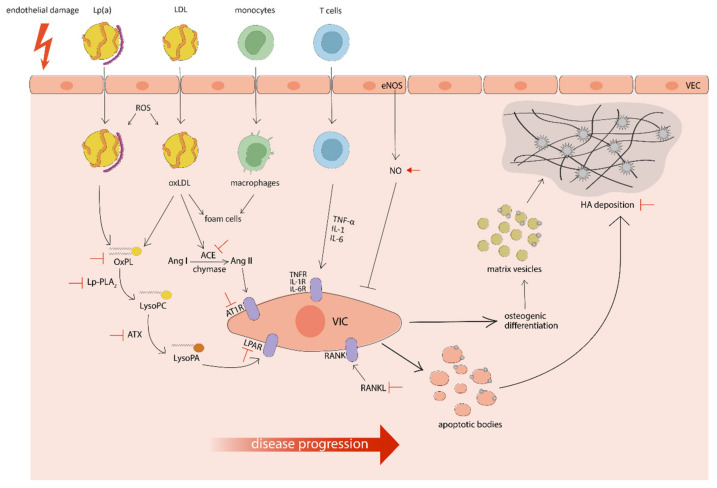
A simplified schematic representation of the pathophysiology of calcific aortic valve stenosis with targets under investigation for medical treatments and potential interventions (in red). The regular arrow means “activation”, the T-shaped arrow stands for “inhibition”.

Valvular interstitial cells (VICs) distributed throughout the three extracellular matrix (ECM) layers are the predominant population of cells in the aortic valve (AV) and play a role in the progression of CAVS [10]. The tissue is surrounded by a monolayer of valvular endothelial cells (VECs), which also seem to be involved in disease development [11]. Physiologically, VICs are in a quiescent state, but they can be activated by transforming growth factor β (TGF-β) and pro-inflammatory cytokines to a myofibroblast-like phenotype [12]. The activation of VICs leads to an increased production and deposition of the ECM components, in particular collagen fibers. The excessive accumulation of disorganized collagen fibers results in fibrotic remodeling of the tissue, increasing the stiffness of the leaflets [1]. In this phase, VICs can also undergo apoptosis, releasing apoptotic bodies that act as nucleation sites for microcalcification [13]. Another important process of the propagation phase is valvular calcification, promoted by two different mechanisms: dystrophic calcification and biomineralization. The first process consists of a passive deposition of amorphous hydroxyapatite (HA) crystals, composed of calcium and phosphate ions, on apoptotic bodies and in the degraded ECM. The second process is similar to skeletal bone formation and is driven by the osteogenic differentiation of VICs, promoted by several signaling pathways including RANK/RANKL [14], ENPP1 [15], and Wnt/β-catenin [16]. The osteoblast-like phenotype, characterized by increased expression of osteogenic markers (such as RUNX2, BMPs, osteocalcin, osteopontin, and bone sialoprotein) [17], can release phosphate- and calcium-rich matrix vesicles, which progressively aggregate and act as scaffolds for HA crystals deposition [18]. In addition, these vesicles contain ectonucleotidases, which generate inorganic phosphate ions from endogenous sources promoting further formation of HA crystals. Concurrent dystrophic calcification and biomineralization result in massive deposition of bone-like minerals in the valvular ECM [19] (see Figure 1).

## 4. Purpose

The development of an effective pharmacological treatment for CAVS is an urgent medical need, considering the prevalence of the disease and the current lack of medical therapies. Several preclinical studies and clinical trials have been performed in recent years to assess the potential beneficial effects of medical treatments. The present review summarizes the current knowledge and latest advances in the medical management of patients with CAVS, placing emphasis on the drug classes with the most relevant and recent preclinical and clinical data, including lipid-lowering interventions, vasoactive drugs, and anti-calcific agents. In addition, the physiopathology of CAVS has been deepened in recent years and several novel targets have been discovered. Among them, the present review reports the targets with preclinical evidence for the development of innovative therapeutic approaches.

## 5. Lipid-Lowering Interventions

### 5.1. Statins

#### 5.1.1. Current Evidence

Statins are 3-hydroxy-3-methylglutaryl coenzyme A (HMG-CoA) reductase inhibitors that reduce the hepatic synthesis of cholesterol, lowering its intracellular levels. In response to the reduction in intracellular cholesterol, LDL receptor (LDL-R) expression increases on hepatocytes, enhancing LDL uptake and decreasing LDL cholesterol (LDL-C) plasma levels. Statins are prescribed in the primary and secondary prevention of atherosclerotic disease, where their effectiveness and established benefits are attributed to pleiotropic effects such as anti-inflammatory response, improved endothelial function, and increased plaque stability [20]. For the similarities in the pathophysiology of atherosclerosis and CAVS, pharmacological strategies effective in atherosclerosis might also modulate CAVS progression.

Statins have been shown to prevent osteogenic differentiation and to reduce calcium deposition in cultured human VICs [21]. In addition, atorvastatin administration reduces AV calcification and expression of osteogenic markers in an animal model of CAVS [22].

Several retrospective studies, involving patients with various severity of CAVS, associated statin treatment with slower progression of CAVS [23,24]. These first encouraging results were confirmed by the RAAVE trial, a prospective nonrandomized study that showed the beneficial effects of statin therapy for patients with asymptomatic moderate to severe CAVS, providing rationale for multiple trials [25]. Unfortunately, none of the randomized controlled trials (SALTIRE, TASS, ASTRONOMER, PROCAS) supported the hypothesis that statin treatment would reduce the progression of CAVS [26,27,28,29]. In the SEAS trial, a long-term co-administration of simvastatin and ezetimibe did not halt CAVS progression or the risk of associated cardiovascular (CV) events [30]. TASS, PROCAS, and SALTIRE might have been too small (less than 200 patients) and with too short follow-up (2 years) for conclusive results. On the other hand, SEAS and ASTRONOMER, larger trials with longer follow-up (4 years) and enrolling of patients (respectively, 1873 and 269) with various severity of CAVS, were powered enough to obtain significant results but they did not detect any treatment effects [31]. A meta-analysis of the above-reported clinical trials concluded that the protective effect of statins in CAVS patients was detected by low-quality (retrospective or nonrandomized) studies, but it was not confirmed by high-quality (prospective or randomized) studies, where the treatment failed to show any significant improvement in clinical outcomes [32]. More recently, a post hoc analysis of three large-scale clinical trials (TNT, IDEAL, and SPARCL) has observed that high-dose atorvastatin treatment for patients without known CAVS does not reduce the incidence of clinical disease compared with low-dose statin or placebo [33]. Taken together, clinical data have clearly shown the lack of efficacy of a lipid-lowering therapy with statins in halting disease progression or preventing the onset. As a consequence of the negative findings, the prescription of statins is not recommended for the treatment or prevention of CAVS in the American and European guidelines [34,35].

#### 5.1.2. Future Perspectives

Recently, a secondary analysis of the SEAS trial has correlated simvastatin–ezetimibe treatment with slower disease progression and reduced need for AVR only in patients with mild CAVS and high pre-treatment LDL cholesterol levels [36], suggesting the conditions for an effective lipid-lowering therapy. It is possible that previous findings failed to show beneficial effects due to the enrolment of patients with well-established CAVS, in whom the disease was too advanced or the atherosclerotic-like process was not the central mechanism of disease progression. Moreover, an in vitro study detected a pro-calcific effect of statins if administered in differentiated VICs, further supporting the hypothesis that the efficacy of the treatment is dependent on the stage of CAVS [37].

As suggested by a recent systematic review, large and high-quality trials should first of all identify patients at high risk for developing CAVS and then, investigate whether early statin treatment can prevent disease progression in these subjects [38]. Currently, the BICATOR trial (NCT02679261) is investigating whether atorvastatin is effective in reducing CAVS progression in patients with bicuspid AV and moderate valve dysfunction [39]. If successful, it could provide a rationale in the use of statins for the prevention of CAVS or the treatment of AV sclerosis.

### 5.2. Lp(a)-Lowering Therapies

#### 5.2.1. Current Evidence

Lipoprotein(a) (Lp(a)) is a cholesterol-rich particle composed of an LDL-like moiety and an apolipoprotein(a) (apo(a)) covalently bound to an apolipoprotein B-100 (apo-B100) through a disulfide bond. Lp(a) acts as atherogenic and prothrombotic lipoprotein but the mechanisms responsible for its pathogenicity are poorly understood. Probably, as an LDL-like particle, Lp(a) secreted in circulation can infiltrate the endothelium and accumulate in arterial and AV subendothelium, where it is recognized by LDL-R and provides a substrate for inflammation and lipid peroxidation [40].

In recent years, the association between Lp(a) and CAVS development has been deeply assessed in several experimental and clinical studies. Lp(a) and its associated pro-inflammatory molecules, including apo(a), oxidized phospholipids (oxPLs), and autotaxin (ATX), are overexpressed in the plasma and AV leaflets of patients with mild to moderate CAVS [41]. A treatment with Lp(a) has been shown to induce VICs osteogenic differentiation, apoptosis, calcium deposition, and ROS production, demonstrating in vitro the causal relationship between Lp(a) and CAVS development [42].

Plasma Lp(a) concentration is primarily (more than 90%) genetically determined by variation in the *LPA* locus, which encodes the apo(a) component of Lp(a) in the hepatocytes. In particular, the two single nucleotide polymorphisms (rs10455872 and rs3798220) responsible for genetically increased Lp(a) levels have been strongly associated with AV calcification, risk of coronary artery disease, and clinical CAVS, reaching genome-wide significance [43,44]. Very recently, Lp(a) levels have been correlated to AV microcalcification in patients without known CAVS and it has been proposed to measure this parameter in subjects with high Lp(a) levels to detect early events of CAVS before the onset of clinically manifested disease [45]. Furthermore, Lp(a) levels have been linearly associated with faster CAVS progression and increased need for AVR in patients with pre-existing mild to moderate CAVS [46] and in elderly patients with advanced CAVS [42], providing stronger rationale for targeting Lp(a). Taken together, these findings support the need to measure plasma Lp(a) concentration for identifying subjects at higher risk for CAVS and in CAVS patients for surveillance of progression, as suggested by several guidelines [47,48]. The measurement of Lp(a) or genetic diagnosis should be done to better define subjects who are more likely to benefit from specific Lp(a)-lowering drugs.

There is a lack of pharmacological agents that specifically lower Lp(a) levels. Statin treatment does not lower plasma Lp(a) concentrations and might even raise them [49]. A non-specific Lp(a)-lowering treatment is niacin, which has been associated to a significant mean Lp(a) reduction of about 23% in a meta-analysis of randomized trials [50]. Despite its established effectiveness in lowering lipid levels, treatment with extended-release niacin has failed to show any clinical benefit in patients with atherosclerosis and background statin therapy, while it increases the frequency of serious adverse effects [51,52]. Thus, niacin therapy is not considered the optimal Lp(a)-lowering therapy and is not recommended by the latest guidelines [47].

A more specific approach to target Lp(a) is the RNA-targeted therapy, which employs small interfering RNA (siRNA) and antisense oligonucleotides (ASOs) to halt Lp(a) hepatic synthesis. In recent years, three ASOs targeting hepatic *LPA* RNA have been developed to specifically reduce apo(a) production and Lp(a) assembly: ISIS-APO(a)_Rx_, IONIS-APO(a)-L_Rx_, and AKCEA-APO(a)-L_Rx_. In recent preclinical and clinical studies, they have been shown to lower human Lp(a) and apo(a) levels in a selective, potent, and dose-dependent manner, suggesting a novel effective, tolerable, and specific Lp(a)-lowering therapy [53].

The precise mechanism by which Lp(a) promotes CAVS progression is still poorly defined but there is growing evidence that its effect is determined by the ability to deliver oxPLs to the AV [54]. OxPLs on Lp(a) are present in the lipid phase of Lp(a) as well as covalently bound to apo-B100 and apo(a) [55]. Elevated plasma levels of oxPLs have been correlated to the presence and progression of atherosclerosis, higher risk of CV events, and increased incidence of stroke, providing strong evidence that oxPLs are major contributors to the pathogenicity of Lp(a) [56]. In addition, high oxPLs levels have been strongly associated with faster CAVS progression and more frequent need for AVR in patients with pre-existing CAVS [57]. Taken together, these data confirm the hypothesis that Lp(a) mediates pro-calcific and pro-inflammatory responses in CAVS through its oxPLs content. OxPLs play an important role in the uptake of lipoproteins by macrophages: in particular, macrophage scavenger receptors recognize the phosphocholine headgroup of oxidized but not native phospholipids. This epitope is also specifically recognized by the IgM monoclonal antibody E06, which inhibits osteogenic differentiation of VICs [42]. In vivo, its expression has been correlated with reduced atherosclerotic development and decreased AV calcification [58], providing an effective possibility to reduce the pro-inflammatory potential of oxPLs. These preliminary data suggest that novel therapies targeting oxPLs may be useful in slowing CAVS progression, but more preclinical research is needed to test this hypothesis.

OxPLs on Lp(a) can be hydrolyzed into oxidized free fatty acid and lysophosphatidylcholine (LysoPC), a strong pro-inflammatory molecule that promotes osteoblastic differentiation of human VICs [59]. LysoPC can be further hydrolyzed in lysophosphatidic acid (LysoPA) by ATX, a lysophospholipase D that is transported in the AV by apo(a) or apoB of Lp(a) and is also secreted by VICs [60]. There is recent evidence of LysoPA and ATX involvement in the pathogenesis of CAVS. ATX, overexpressed in stenotic AVs and more enzymatically active in patients with CAVS, can be a predictor of the disease [61]. LysoPA, generated by ATX, has been shown to promote the osteogenic differentiation of cultured VICs and to favor the development of CAVS in a mouse model [60]. LysoPA/ATX signaling axis could represent a novel target and may be halted at different levels through ATX inhibitors or by blocking the LysoPA-induced pathway. The first-in-class ATX inhibitor GLPG1690 has displayed safety, tolerability, and good pharmacokinetic profile in healthy subjects [62] and preliminary efficacy in patients with idiopathic pulmonary fibrosis [63], but its safety and efficacy have not been assessed in patients with CV diseases. LysoPA seems to exert pro-calcific effects by binding on VICs’ surfaces to its receptor LPAR1, overexpressed in stenotic AVs, which activates the NF-κB/IL-6/BMP-2 pathway [64]. Pharmacological blockade of LPAR1 with the receptor antagonist Ki16425 prevents the LysoPA-mediated mineralization of cultured VICs [60] and platelet-induced mineralization of the AV in a mouse model [65]. Taken together, these data suggest that blocking LPAR1 could represent a novel strategy for CAVS that warrants further research.

The enzyme responsible for the hydrolysis of oxPLs into LysoPC is the lipoprotein-associated phospholipase A2 (Lp-PLA_2_), carried in the plasma by LDL and Lp(a) and increased in stenotic AVs [59]. Increased Lp-PLA_2_ activity has been correlated to faster progression of CAVS in patients with mild CAVS, providing evidence for Lp-PLA_2_ involvement in the early development of the disease and a rationale for randomized trials targeting Lp-PLA_2_ activity [66]. The inhibition of Lp-PLA_2_ with darapladib might be a valuable approach for patients in early stages of CAVS. Darapladib has proven to slow the development of atherosclerosis in animal models [67], but its anti-atherogenic effect has not been confirmed in randomized trials [68]. Although the pathophysiological processes of atherosclerosis are similar to CAVS, these findings cannot be directly transposed and proper investigations on CAVS patients are required to assess whether Lp-PLA_2_ inhibition could be considered a novel therapeutic strategy for CAVS.

#### 5.2.2. Future Perspectives

Several different strategies for reducing Lp(a) levels have been detected and could be further investigated, including the targeting of *LPA*, oxPLs, ATX, LPAR1, and Lp-PLA_2_. Even the PCSK9-lowering strategies could be used to reduce Lp(a) levels. However, it remains unclear whether the lowering of Lp(a) can reduce the risk of developing CAVS and improve CV outcomes in subjects with elevated plasma Lp(a) concentration. Thus, future trials should focus on this fundamental aspect.

Currently, the ongoing EAVaLL trial (NCT02109614) is investigating whether a treatment with extended-release niacin in patients with mild CAVS and elevated Lp(a) could lower plasma Lp(a) levels and slow disease progression [69] (see Table 1). If successful, this study could be the first step for trials with other Lp(a)-lowering treatments. A more specific approach for lowering Lp(a) levels is now in development: AMG890, a first-in-class candidate siRNA which targets *LPA* RNA. Its safety, tolerability, and efficacy are now being assessed in a phase I trial (NCT03626662) involving healthy volunteers with elevated levels of Lp(a) [70] and in a phase II trial (NCT04270760) in patients with atherosclerotic CV disease [71].

### 5.3. PCSK9 Inhibitors

#### 5.3.1. Current Evidence

Proprotein convertase subtilisin/kexin type 9 (PCSK9) is a circulating plasma protein primarily produced and secreted by the liver that promotes LDL-R lysosomal degradation, decreasing its density on hepatocytes’ surfaces. LDL-R is responsible for the internalization of LDL and Lp(a) and its depletion increases plasma levels of these lipoproteins. An increase in PCSK9 plasma levels, due to mutations in the *PCSK9* gene and to pathophysiological determinants [72], causes dyslipidemia and hypercholesterolemia, risk factors for the development of CV diseases [73]. The correlation between PCSK9 levels and CAVS has been investigated in recent years: a cross sectional study has detected an increase in plasma PCSK9 concentration in patients with CAVS, in particular in the early stage, finding a correlation between circulating PCSK9 levels and the presence but not severity of the disease [74]. PCSK9, infiltrated from the blood stream and locally secreted by VICs, is significantly increased in human calcified AVs, where it correlates positively with the extent of calcification [75]; conversely, PCSK9 deficiency reduces the calcification potential [76]. These recent studies have found a direct effect of PCSK9 on the development and progression of CAVS, providing a rationale for a therapeutic strategy based on its inhibition. PCSK9 can be inhibited at the protein level with specific antibodies or at the RNA level by targeting *PCSK9* RNA with siRNA.

Evolocumab and alirocumab, humanized monoclonal anti-PCSK9 antibodies, have been designed to increase LDL-R on hepatocytes, reducing LDL and Lp(a) plasma concentration. In vitro, treatment with anti-PCSK9 antibody significantly reduces calcium accumulation in VICs cultures [75]. There is growing clinical evidence that the subcutaneous administration of alirocumab and evolocumab in statin-intolerant patients is a safe, tolerable, and effective treatment for hypercholesterolemia, leading to great and persistent reduction in LDL-C levels [77,78]. Recently, the FOURIER trial has demonstrated that the addition of evolocumab to background statin therapy decreases the risk of CV events in patients with established atherosclerotic CV disease, lowering LDL-C levels by at least 30% in 99% of subjects and Lp(a) levels by a median of 27% [79,80]. In the FLOREY trial involving healthy men, evolocumab has proven to lower plasma Lp(a) levels up to 36% with two mechanisms: by decreasing Lp(a) production as monotherapy and by increasing Lp(a) catabolism in combination with atorvastatin [81]. However, analysis of the plaque composition has demonstrated that evolocumab does not change the calcium volume of coronary atherosclerotic plaques [82]. Recently, the ODYSSEY OUTCOMES trial has detected a reduction in the risk of major adverse CV events after addition of alirocumab to background statin therapy in patients with recent acute coronary syndrome [83], confirming the additional beneficial effect of this lipid-lowering therapy.

#### 5.3.2. Future Perspectives

The above-described recent data focus on the capability of PCSK9-lowering treatments to reduce lipoproteins levels, but clinical evidence evaluating their potential effects on patients with CAVS is lacking. Currently, a randomized, placebo-controlled trial (NCT03051360) is assessing whether the administration of an anti-PCSK9 antibody to patients with mild to moderate CAVS can slow disease progression by decreasing Lp(a) and LDL-C levels [84] (see Table 1). If successful, it could support the design of other trials to further investigate this pharmacological therapy.

Another novel approach to lower PCSK9 concentration is the blocking of hepatic synthesis with inclisiran, a siRNA targeting *PCSK9* RNA. Inclisiran, administered subcutaneously every 6 months, is an effective PCSK9- and LDL-lowering treatment with good patient tolerability [85]. Very recently, three pivotal phase III trials (ORION-9, ORION-10, ORION-11) have detected a persistent reduction in LDL-C levels by approximately 50% with inclisiran in patients with atherosclerotic CV disease or familial hypercholesterolemia [86,87]. These promising results provide a rationale for clinical trials involving CAVS patients to assess whether this lipid-lowering treatment can reduce disease progression.

## 6. RAAS Blockade

### 6.1. ACE Inhibitors/ARBs

#### 6.1.1. Current Evidence

Systemic arterial hypertension is a frequent comorbidity in CAVS, affecting at least 30% of symptomatic patients [88]. High systemic pressure increases the mechanical forces, favoring an endothelial lesion in the aortic side of the leaflets, and alters the expression of markers involved in inflammation (VCAM-1, IL-6), ECM remodeling (MMPs, collagen), and calcification (osteopontin) [89], increasing the progression rate of CAVS. The stenotic valve and the systemic vascular resistance represent two resistances in series, both contributing to pressure overload in the left ventricle (LV) [90]. In patients with CAVS, hypertension increases LV hypertrophic remodeling and afterload, resulting in higher incidence of clinical CV events [91]. Treating hypertension could be useful to improve the clinical outcomes of CAVS patients. Traditionally, an antihypertensive treatment was contraindicated because it would decrease systemic vascular resistance without increasing cardiac output, with the risk of severe hypotension and lower myocardial perfusion [92]. However, recent trials have reported no change in heart rate or evident adverse effects in patients with CAVS treated with antihypertensive treatments [93]. To date, the most studied class of antihypertensive for the management of hypertensive patients with CAVS is the renin–angiotensin–angiotensinogen system (RAAS)-blocking therapy.

RAAS regulates arterial blood pressure at different levels. Angiotensinogen is hydrolyzed by renin into the inactive precursor angiotensin I, further hydrolyzed by the angiotensin-converting enzyme (ACE) into the active angiotensin II (Ang II). In addition to ACE, the enzyme chymase is involved in the production of Ang II. Ang II binds to its Ang II type 1 receptor (AT1R) inducing inflammation, oxidative stress, fibrosis, and vasoconstriction [94]. In addition, ACE promotes the inactivation of bradykinin, required for the synthesis of NO, limiting its vasodilator effect. RAAS overactivation leads to hypertension; its blockade through ACE inhibitors (ACEI) or Ang II receptor blockers (ARBs) is a common antihypertensive strategy. There is evidence of RAAS involvement in CAVS progression: Ang II has been shown to increase IL-6 production, to promote cardiac fibrosis, and to induce osteogenic differentiation of cultured VICs [95,96]. Human stenotic AVs are characterized by an overexpression of chymase, AT1R, and ACE, providing evidence of a local activation of RAAS in the valve [97]. Moreover, ACE is colocalized with lipoproteins in the valve lesions, suggesting that ACE is delivered in the AV by LDL [98]. Based on these recent findings, the blockade of RAAS could give cardioprotective and beneficial effects; clinical trials with ACEI and ARBs have been performed to test the hypothesis.

ACEI therapy was well tolerated in patients with various severity of CAVS [99,100] and its effects were assessed in some small-size trials. The first one failed to show any beneficial hemodynamic effect of ACEI in monotherapy or in combination with statins [23]. In contrast, ACEI were associated with lower AV calcification, hemodynamic benefits, and slower progression of mild CAVS in consequent trials [101,102]. More recently, two small randomized trials (ACCESS and RIAS), involving patients with moderate to severe CAVS, have detected hemodynamic improvement and progressive reduction in LV hypertrophy with ACEI [103,104]. These studies were not powered enough for relevant clinical outcomes; therefore, the preliminary promising findings should be confirmed by larger clinical trials.

ARBs were tested in patients with severe CAVS because AT1R are activated and involved in the AV remodeling and fibrosis, a later stage of CAVS. In the prospective ROCK-AS trial, candesartan was well tolerated but failed to show efficacy [105]. In contrast, two consequent studies associated the treatment with ARBs (but not with ACEI) to a reduction in AV remodeling, inflammation, IL-6 expression, and fibrosis score, suggesting that ARBs could halt the fibrotic process by lowering tissue inflammation [106,107]. It is likely that a large amount of Ang II is produced by chymase instead of ACE and the most effective way to reduce AV remodeling is the downstream blocking of AT1R through ARBs.

The effect of RAAS blockade with ACEI and ARBs was also investigated in larger trials with promising findings. An observational population-based study correlated ACEI and ARBs with higher survival and lower risk of CV events in patients with various degrees of CAVS [108]. A large retrospective study of patients included in the SEAS trial confirmed previous data, detecting that RAAS blockade did not increase the risk of CV and all-cause mortality and reduced LV mass progression [109]. The JAAS prospective analysis concluded that ACEI and ARBs may be beneficial before or early after the development of CAVS [110].

#### 6.1.2. Future Perspectives

The above-described studies, reporting conflicting results, only partially demonstrated the efficacy of ACEI and ARBs in reducing AV remodeling and CAVS progression. Currently, two clinical trials are ongoing to further investigate the potential beneficial effects of ARBs, in particular fimasartan (ALFA trial, NCT01589380) and losartan (NCT03666351), in hypertensive patients with moderate to severe CAVS [111,112]. However, these studies evaluate the change in hemodynamic parameters and LV remodeling; they are not specifically designed to determine the effects of the treatment on valvular calcification.

Further large-scale randomized trials are required to determine whether a RAAS-blocking therapy can delay the need for AVR, halt disease progression, and lower the mortality rate in patients with moderate to severe CAVS, as suggested by a recent review [113]. The current American and European guidelines recommend treating coexisting systemic hypertension in patients with asymptomatic CAVS or at high risk for developing CAVS by choosing an antihypertensive drug that avoids hypotension [34,35], but they do not specify which treatment should be used. To date, trials are missing on different classes of antihypertensive drugs, the RAAS-blocking therapies represent the treatment of choice, and the above-reported studies suggest targeting RAAS not as a CAVS-specific treatment, but for treating CAVS-related comorbidities.

In recent years, several trials have been performed to assess the effects of a postoperative RAAS blockade on clinical outcomes. Post-AVR treatment has been associated with improved long-term outcomes [114] and a lower risk of all-cause and CV mortality [115,116]. The global cardiovascular protective effect might be partially explained by a positive LV remodeling; a randomized trial (ARISTOTE, NCT03315832) is ongoing to confirm this hypothesis [117]. Taken together, these recent clinical data suggest treating patients who underwent AVR with a postoperative RAAS-blocking therapy to improve the prognosis. Based on this evidence, a RAAS-blocking therapy seems to be more effective if prescribed after surgical or transcatheter AVR.

### 6.2. Mineralocorticoid Receptor Antagonists

#### 6.2.1. Current Evidence

An alternative approach to reduce RAAS activation is the administration of mineralocorticoid receptor antagonists (eplerenone and spironolactone), used for the treatment of hypertension. Spironolactone has been shown to prevent vascular calcification in vitro and to improve CV clinical outcomes [118]; however, its effects have never been assessed in clinical trials involving patients with CAVS. Eplerenone has proven to halt the process of vascular calcification in vitro [119] and to exert an anti-atherosclerotic effect in vivo [120]. Moreover, it reduces LV hypertrophy in hypertensive subjects [121] and improves clinical outcomes among patients with systolic heart failure [122]. A study in hypercholesterolemic rabbits has shown the presence of mineralocorticoid receptors in the AV and has reported the beneficial effects of a selective antagonism with eplerenone in the early stage of CAVS [123]. Despite the promising preclinical findings, the only clinical trial involving CAVS patients has failed to show efficacy of eplerenone in reducing CAVS progression or LV dysfunction [124].

#### 6.2.2. Future Perspectives

To our knowledge, no ongoing studies have been designed to evaluate the potential effects of the mineralocorticoid receptor antagonists on the progression of CAVS. An early phase I trial (NCT03923530) is currently investigating whether a postoperative eplerenone treatment can improve the outcomes and the quality of life among patients with hypertension and abnormal hemodynamics after TAVR [125]. In the case of positive outcomes, it would confirm the hypothesis that RAAS blockade is more effective if administered after AVR. However, it remains unclear and to be evaluated in future studies whether the modulation of the mineralocorticoid receptor could give a protective effect on the progression of CAVS.

## 7. Modulators of Nitric Oxide Pathway

### 7.1. Current Evidence

Nitric oxide (NO) is a potent vasodilator mainly produced by endothelial NO synthase (eNOS) and secreted by endothelial cells. Endothelial-derived NO binds to the heme group of intracellular soluble guanylyl cyclase (sGC), increasing the production of cGMP. The activation of the NO/sGC/cGMP pathway plays a central role in many physiological mechanisms, such as vasodilation, inhibition of platelet aggregation, anti-inflammatory, and anti-fibrotic processes. In the aortic valve, NO released by VECs prevents matrix calcification and osteoblastic differentiation of VICs, exerting protective effects [126]. In pathological conditions, eNOS downregulation and uncoupling lead to increased ROS production, enhanced oxidative stress, and decreased NO bioavailability, promoting the progression of CAVS [127].

NO donors, currently used for the management of acute heart failure, could offer an advantage in patients with severe CAVS and concomitant congestive heart failure or LV dysfunction, a high-risk population with worse clinical outcomes and higher mortality rate. Although conventionally, they were contraindicated in CAVS due to the risk of severe hypotension, they could be a useful treatment for patients with cardiac comorbidities for their ability to reduce LV consumption of oxygen, filling pressure and afterload, and improving cardiac perfusion and functionality [91].

In addition to the effects on the vasculature, NO donors seem to act directly in the valve; for example, L-Arginine and nitroprusside have been shown to inhibit osteogenic differentiation of cultured VICs [128,129]. This anti-calcific effect seems to be mediated by sGC activators (such as BAY and YC-1) or by agents increasing intracellular cGMP levels, while it is prevented by sGC inhibitors (such as ODQ), suggesting that the protective action of NO on VICs is probably dependent on the activation of the sGC/cGMP pathway [130,131]. Collectively, these findings motivate potential therapeutic strategies based on the activation of the NO/sGC/cGMP pathway, providing a rationale for clinical trials with NO donors and sGC activators to test this hypothesis.

To date, the effects of NO donors have been assessed in some small-size trials involving patients with severe CAVS and concomitant CV diseases. These studies detected an improvement in cardiac output and a beneficial decrease in LV afterload and filling pressure without increasing the risk of hypotension [132,133]. However, the aim of the trials was the management of patients with heart failure and concomitant CAVS; they were not designed to assess the effects of NO donors on CAVS progression and future studies should investigate these aspects.

Another pharmacological approach to activate the NO/sGC/cGMP pathway is the inhibition of phosphodiesterase type 5 (PDE5), the enzyme responsible for hydrolysis of cGMP to GMP. The PDE5 inhibitor sildenafil, an oral vasodilator widely used for erectile dysfunction, is also effective for pulmonary arterial hypertension, where PDE5 is upregulated [134]. The potential beneficial effects of PDE5 inhibition for the treatment of CV diseases have been investigated in several trials. At the cardiac level, sildenafil improves hemodynamic parameters, cardiac output and LV diastolic function in patients with heart failure [135]. An overexpression of myocardial PDE5 has been also observed in patients with severe CAVS or LV dysfunction [136] and its inhibition could provide a beneficial effect, but to date, only two small-size trials have been performed on CAVS patients. The first pilot study (ASPEN trial, NCT01275339), involving subjects with moderate to severe CAVS, was terminated without results for difficulty of enrolling patients [137]. In the second trial, a short-term treatment with sildenafil to patients with severe symptomatic CAVS was safe, well tolerated, and effective in improving hemodynamic parameters [138]. However, the study was not designed to evaluate the effects of the treatment on CAVS progression.

### 7.2. Future Perspectives

The above-reported clinical data provided evidence for the safety of NO donors and PDE5 inhibitors in CAVS, but no convincing data are available on their long-term use and clinical efficacy. In addition, these trials were specifically focused on the change in hemodynamic parameters and LV remodeling. Thus, long-term and larger randomized trials are required to confirm preliminary findings and future studies should investigate the effects of the treatments on CAVS progression.

Concerning sGC activators, a potential candidate is ataciguat (HMR1766), which preferentially binds to the oxidized state of sGC, increasing its enzymatic activity independently of NO release. Ataciguat induces NO production in endothelial cells [139], improves vascular functions in an animal model of heart failure [140], and reverses hemodynamic changes in experimental pulmonary hypertension [141]. The safety and effects of ataciguat are currently being assessed in two studies involving patients with mild to moderate CAVS. The first trial (NCT02049203) is evaluating the safety of the treatment [142]; the second one (NCT02481258) aims to determine the long-term efficacy in reducing LV dysfunction and slowing disease progression [143] (see Table 1). If successful, they could provide a rationale for larger randomized trials.

## 8. Anti-Calcific Agents

The calcification of the valve plays a central role in driving disease progression and can represent a key target for novel therapies. The mechanisms involved in valvular calcification are similar to those of skeletal bone formation and inhibitors of pathological mineralization (bisphosphonates, denosumab, and vitamin K) could provide an effective strategy to reduce ectopic calcification. Their effects are being assessed in experimental studies and clinical trials.

### 8.1. Inhibitors of Bone Resorption

#### 8.1.1. Current Evidence

Bisphosphonates and denosumab are pharmacological treatments widely used for bone diseases characterized by excessive osteoclast-mediated bone resorption, such as osteoporosis. Bisphosphonates are inhibitors of osteoclast differentiation and activity on bone surface; denosumab is a human monoclonal antibody that targets the receptor activator of nuclear factor kappa B ligand (RANKL), avoiding its binding to its transmembrane receptor RANK on different cell types. In bone, RANK activation on pre-osteoclasts’ surfaces induces their differentiation to osteoclasts, increasing the availability of calcium and phosphate ions in the blood. The process can be counteracted by osteoprotegerin (OPG), a soluble decoy that targets RANKL preventing RANK activation. There is growing evidence of the local activation of the OPG/RANK/RANKL pathway in the CV system and AV in particular. OPG exerts a protective effect in the vasculature and AV [144], confirmed by the fact that OPG-deficient mice develop osteoporosis and vascular calcification [145]. Calcified arteries and stenotic AVs are characterized by decreased expression of OPG and increased levels of RANKL [146]. There is recent evidence of an upregulation of RANKL expression in circulating mononuclear cells of patients with CAVS, favoring ectopic calcification. In addition, the treatment with RANKL promotes matrix calcification and osteoblastic differentiation of cultured cells [14,147]. Based on these data, RANK seems to be also expressed on VICs’ surfaces, where its activation promotes CAVS progression.

Several cross-sectional and longitudinal studies have reported a strong inverse correlation between bone mineral density and valvular/vascular calcification [148,149]. This phenomenon, which is referred to as the “calcification paradox”, may be explained by common pathways with reciprocal effects on bone and vasculature simultaneously (including the OPG/RANK/RANKL axis) and suggests that treatments for osteoporosis might have a beneficial effect on vascular/valvular calcification while maintaining appropriate bone density.

Concerning denosumab, only preclinical data are available. Denosumab reduces vascular calcification in glucocorticoid-induced osteoporotic mice [150] and inhibits calcium deposition in porcine VICs cultures [151].

Bisphosphonates were developed as more stable analogues of inorganic pyrophosphate and their effects have been investigated in several preclinical and clinical studies. They have been shown to prevent the osteogenic differentiation of cultured cells [152] and to inhibit valvular/vascular calcification in mice at doses comparable to those used for osteoporosis [153]. In a recent study on an experimental model of CAVS, local delivery of zoledronate at the level of AV has shown safety and efficacy in inhibiting the progression of AV calcification [154]. In addition to reducing bone resorption, the beneficial effects of bisphosphonates in the CV system could be related to the extra-skeletal properties, including the decrease in cytokines secretion, the downregulation of adhesion molecules, and the reduction in circulating monocytes and LDL-C [155]. Several retrospective studies investigated the extra-skeletal effects of bisphosphonates. The first small-size clinical trials, involving dialysis patients with end-stage renal disease and concomitant CV calcification, detected a decrease in arterial calcification after treatment with etidronate, improving long-term outcomes [156,157]. Several recent trials have assessed the effects of bisphosphonates on CV calcification: a large multi-ethnic study (MESA) associated the bisphosphonate treatment with decreased prevalence of CV calcification in older women with subclinical CV diseases [158]; some observational retrospective studies reported an increase in AV area and slower disease progression in patients with CAVS treated with bisphosphonates [159,160]. In contrast, a large retrospective study involving older women with mild to moderate CAVS did not observe any significant impact of bisphosphonates on the progression of the disease [161].

#### 8.1.2. Future Perspectives

The main limitation of the above-reported studies was the prescription of bisphosphonates only to patients with concomitant osteoporosis, which has an additional effect on the development of CAVS and could be a major confounder [162]. Prospective data are lacking and the potential efficacy of the treatment should be further evaluated in large randomized controlled trials involving patients without bone diseases. Currently, the ongoing SALTIRE II trial (NCT02132026) has randomized 150 patients with mild CAVS and no concomitant osteoporosis to denosumab, alendronate, or placebo [163] (see Table 1). If successful, it will be the first study to determine whether osteogenic drugs can reduce disease progression in patients without bone diseases. The study has been completed and is currently in analysis.

### 8.2. Vitamin K

#### 8.2.1. Current Evidence

Vitamin K is a liposoluble vitamin naturally found as vitamin K1 (phylloquinone) in vegetables and vitamin K2 (menaquinones) in fermented food. Vitamin K serves as a cofactor for the activation of vitamin K-dependent proteins, including matrix Gla protein (MGP), through the γ-carboxylation of Glu residues. The active form of MGP functions as potent inhibitor of soft-tissue calcification by binding calcium ions and avoiding the formation of HA crystals [164]. Active MGP can also interact with bone morphogenetic protein-2 (BMP-2), reducing its osteogenic activity [165]. A beneficial anti-calcific role of MGP in the CV system is supported by experimental evidence: inhibition of *MGP* gene induces osteoblastic differentiation of human VICs [166] and *Mgp*-deficient mice develop arterial calcification [167]. MGP expression was significantly reduced in VICs isolated from stenotic valves [168]. MGP activity can be modulated by vitamin K and its antagonists: a low intake of vitamin K or the assumption of vitamin K-antagonists (warfarin) lead to vitamin K-insufficiency and therefore, lower activation of MGP [169]. There is preclinical and clinical evidence that vitamin K-antagonists accelerate osteoblastic differentiation of cultured VICs, increase vascular/valvular calcification, and promote AV degeneration [170,171]. These findings suggest that vitamin K-independent oral anticoagulants (such as thrombin inhibitors or factor Xa inhibitors) are preferable for patients with early CAVS.

The supplementation of vitamin K has been proposed as a novel strategy to reduce CV calcification by enhancing MGP activity. Vitamin K has also proven to exert an anti-inflammatory effect: vitamin K2 reduces the expression of pro-inflammatory cytokines in cell cultures [172] and the dietary supplementation of vitamin K1 has a similar effect in animal models [173]. The MESA study has found an inverse correlation between serum levels of vitamin K1 and circulating inflammation markers, such as ICAM-1, C-reactive protein, and IL-6 [174]. In addition, vitamin K2 decreases cholesterol biosynthesis and increases LDL-R, reducing plasma lipid levels [175]. The anti-inflammatory and lipid-lowering properties of vitamin K, independent of γ-carboxylation, may also contribute to the inhibition of ectopic calcification and provide a rationale for clinical trials with vitamin K supplementation.

The effects of oral vitamin K assumption have been investigated in some prospective randomized controlled trials with conflicting outcomes. In the CACK2 and Vitacal trials, vitamin K2 administration did not decrease the progression of vascular calcification in patients with chronic kidney disease and diabetes [176,177]. These studies were limited by short-term follow up and small sample size. In a long-term trial, daily supplementation of vitamin K1 for 3 years showed beneficial effects in healthy elderly people with pre-existing vascular calcification [178]. An inverse correlation between vitamin K2 intake and risk of coronary heart disease was also detected in two large population-based studies [179,180].

#### 8.2.2. Future Perspectives

The iPACK-HD, VitaVasK, and VitaK-CAC trials are currently assessing the potential efficacy of vitamin K assumption for reducing coronary artery calcification in hemodialysis patients and in subjects with coronary heart disease [181,182,183].

Vitamin K has also been supplemented in recent clinical trials to assess the potential effects on AV calcification. The first randomized controlled trial involving CAVS patients has associated the supplementation of vitamin K1 with slower progression of AV calcification [184]. The ongoing AVADEC (NCT03243890) and BASIK2 (NCT02917525) randomized trials are investigating the effects of vitamin K2 (menaquinone-7) supplementation on the progression of AV calcification, respectively, in patients without clinically significant CAVS or with bicuspid AV and mild to moderate CAVS [185,186] (see Table 1). If successful, they would provide as effective treatment for CAVS a simple, safe, and readily available compound that can be easily supplemented in patients. However, larger phase 3 clinical trials are required to confirm this preliminary hypothesis.

## 9. Emerging Targets

**Figure 2:**
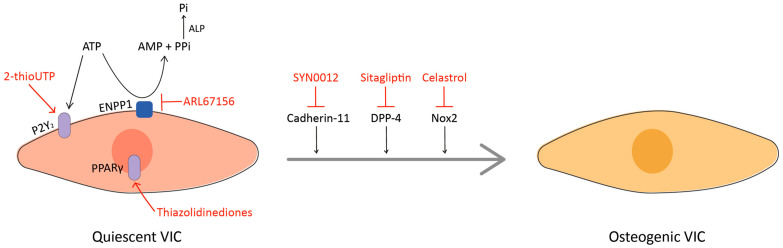
A simplified schematic representation of potential novel targets (in black) and corresponding treatments (in red). The regular arrow means “activation”, the T-shaped arrow stands for “inhibition”.

In recent years, a better understanding of the pathophysiology has led to the identification of several interesting targets for the development of novel therapeutic approaches (see Figure 2). In this chapter, we focused on the role of some innovative targets that have already collected preclinical in vivo evidence (see Table 2). Nevertheless, several other cellular pathways with potentiality for future in vivo application are currently under investigation [187,188,189].

### 9.1. Nox2

NADPH oxidase 2 (Nox2) is a ROS-generating enzyme upregulated in human stenotic AVs and associated with osteoblastic differentiation of cultured VICs [190]. The selective Nox2 inhibitor celastrol is a natural compound used in traditional Chinese medicine for cancer, neurodegeneration, chronic inflammatory, and autoimmune diseases because of its antioxidant property [197]. It also exerts pleiotropic effects, including inhibition of pro-inflammatory cytokines production, reduction in osteoclast formation, and decrease in bone resorption [198]. There is recent evidence that celastrol prevents calcium deposition in cultured VICs and reduces ROS generation, fibrosis, LV remodeling, and calcification without side effects in a rabbit model of CAVS, suggesting the safety and therapeutic effectiveness of the treatment [190]. Despite the promising findings, additional long-term preclinical studies are needed to confirm the potential beneficial properties before entering clinical trials, as suggested by Yeang and colleagues [199].

### 9.2. ENPP1 and P2Y_2_

Ectonucleotide pyrophosphatase/phosphodiesterase 1 (ENPP1) is a membrane-bound member of the ectonucleotidases family, which hydrolyzes extracellular ATP to generate pyrophosphate, a potent inhibitor of vascular/valvular calcification [200]. When ENPP1 is overexpressed, it also increases the levels of inorganic phosphate with a pro-calcific effect [201]. ENPP1 is upregulated in calcific AVs, where it promotes the mineralization of cultured VICs [15]. The pro-mineralizing effect of ENPP1 can be also related to the decrease in ATP levels: in fact, ATP exerts a protective effect on VICs by binding to the purinergic receptor P2Y_2_, which activates the PI3K/Akt pathway [202]. Based on this evidence, inhibitors of ENPP1 or agonists of P2Y_2_ might prevent the mineralization of the valve and the development of CAVS. ARL67156, an inhibitor of ectonucleotidases, and 2-thioUTP, an agonist of P2Y_2_, have been shown, respectively, to prevent disease development and to promote its regression in experimental models of CAVS [191,192], providing evidence that the pathologic mineralization of the valve is a reversible process. The promising preclinical findings should be confirmed in clinical trials to determine the effectiveness of the treatments.

### 9.3. DPP-4

Dipeptidyl peptidase 4 (DPP-4) is a multifunctional enzyme that cleaves the N-terminal dipeptides of several substrates, including chemokines, neuropeptides, and regulatory peptides [203]. DPP-4 expression is increased in the AV after endothelial dysfunction and can induce VICs osteogenic differentiation, while the inhibition of its enzymatic activity with sitagliptin halts the process in vitro [193]. Selective DPP-4 inhibitors, including sitagliptin, are a new class of oral hypoglycemics approved for the treatment of type II diabetes because they avoid the inactivation of glucagon-like peptide 1 (GLP-1) [203]. There is recent evidence that the administration of sitagliptin reduces AV calcification and disease progression in animal models of CAVS [194], suggesting that the repositioning of DDP-4 inhibitors could be a novel strategy for the treatment of CAVS. This hypothesis, further supported by the anti-atherosclerotic and anti-inflammatory properties of DPP-4 inhibitors [204], should be tested in additional preclinical and clinical studies.

The pro-calcific action of DPP-4 seems to be related to the inactivation of insulin-like growth factor-1 (IGF-1), a growth hormone with protective effects on the CV system [205]. There is in vitro evidence of IGF-1 anti-calcific affect in vascular and valvular cells [206], but additional experiments are warranted to confirm its relationship with DPP-4.

### 9.4. Cadherin-11

Cadherin-11 (Cad-11) is a specialized cell–cell adhesion protein related to proper embryonic valve formation and remodeling, but it may also contribute to CAVS pathogenesis if expressed in adulthood [207]. The overexpression of Cad-11 leads to dystrophic calcific nodules in vitro and calcification in mature heart valves by upregulating RhoA and Sox9 expression [208,209]. Conversely, *Cad-11*^-/-^ adult mice do not develop AV calcification [210] and the treatment with SYN0012 (a Cad-11-blocking antibody) reduces calcification, pro-inflammatory signals, and leaflet stiffening in *Notch^+/−^* mice [194]. Taken together, these preclinical data suggest targeting Cad-11 as a novel pharmacological strategy for preventing disease progression in CAVS patients. Very recently, SYN0012 treatment has proven to improve cardiac function and to reduce inflammation-driven fibrotic remodeling of the myocardium in mice, suggesting that blocking Cad-11 might also prevent cardiac fibrosis and heart failure, common comorbidities in CAVS patients [211].

### 9.5. PPARγ

Peroxisome proliferator-activated receptor-gamma (PPARγ) is a nuclear receptor of the PPARs family, regulating the transcription of several genes. The downregulation of PPARγ expression has been associated with atherosclerosis, hypertension, and vascular calcification [212]. Conversely, its activation reduces inflammation, inhibits apoptosis, and improves endothelial function [213], making PPARγ agonists an interesting therapeutic option for the treatment of dyslipidemia, atherosclerosis, and CV diseases. PPARγ agonists are insulin sensitizers for the treatment of type II diabetes, but their use is limited because of safety concerns. Among them, the most used and safer agonist is pioglitazone, which has been shown to reduce lipid deposition, apoptosis, and AV calcification in animal models [195,196], providing a rationale for clinical trials with PPARγ agonists for CAVS treatment. There is recent evidence that the anti-calcific effect of pioglitazone is mediated by the inhibition of the Wnt/β-catenin signaling pathway [214], but more experiments are required to elucidate its mechanism of action on VICs. Although the promising preclinical findings encourage the use of PPARγ agonists for CAVS, the adverse effects (such as bone loss and increased risk of heart failure) limit the application of this therapy [215]. The benefit–risk ratio should be investigated in future preclinical studies.

## 10. Conclusions

Calcific aortic valve stenosis represents an increasing health care burden, but there is no effective pharmacological therapy to avoid the progression towards surgery. Despite some promising data, the above-reported medical treatments have not yet been convincingly shown to slow disease progression or to improve clinical outcomes. Thus, they are currently prescribed for treating CAVS-related comorbidities, not as CAVS-specific treatments. Statins have failed to show effectiveness in halting disease progression or preventing the onset; RAAS blockade has only partially demonstrated efficacy in reducing CAVS progression and RAAS-blocking therapy seems to be more effective if prescribed after AVR. Concerning Lp(a)-lowering therapies, modulators of the NO pathway, and anti-calcific agents, the efficacy of these treatments has not yet been determined in clinical studies or should be further assessed in larger randomized trials. The growing experimental evidence and understanding of the pathophysiology indicate that, although CAVS shares pathophysiological features with atherosclerosis and vascular calcification, it is a specific valvular disease. Moreover, it has been recently revealed that the progression, prognosis, and outcomes of CAVS are sex-dependent; for example, women have lower density of AV calcium, reduced fibrosis, and less inflammatory response compared to men [216,217]. Therefore, novel medical treatments should target valve-specific and sex-related signaling pathways to be more effective. Nowadays, several molecular targets (including Nox2, ENPP1, P2Y_2_, DPP-4, cadherin-11, and PPARγ) are under current investigation for the development of novel therapeutic strategies.

## Figures and Tables

**Table 1:** Ongoing clinical trials involving patients with calcific aortic valve stenosis (CAVS).

Trial Name	NCT	Treatment	Phase	Population	Enrolment (No. of Patients)	Primary Outcome
EAVaLL	NCT02109614	Extended-release niacin vs. placebo	I	Aortic sclerosis or mild CAVS	238	Calcium score progression measured by cardiac CT at 2 years
	NCT03051360	PCSK9 inhibitor vs. placebo	II	Mild to moderate CAVS	140	Calcium score progression measured by cardiac CT and by NaF PET at 2 years
	NCT02481258	Ataciguat vs. placebo	II	Moderate CAVS	35	Changes in AV calcium levels at 6 months
SALTIRE II	NCT02132026	Alendronate/denosumab vs. placebo	II	Peak aortic jet velocity >2.5 m/s and grade 2–4 calcification of the AV on echocardiography	150	Change in AV calcium score at 6 months and 2 years
AVADEC	NCT03243890	Menaquinone-7 vs. placebo	-	AV calcification score above 300, but without clinical CAVS	389	Change in AV calcification at 2 years
BASIK2	NCT02917525	Vitamin K2 vs. placebo	II	Bicuspid AV and mild to moderate CAVS on prior echocardiography	44	Change in AV calcium metabolism measured by NaF PET at 6 months

**Table 2:** Potential novel targets for CAVS and corresponding treatments with in vivo evidence.

Target	Treatment	Effects	Model	Ref.
Nox2	Celastrol	Nox2 inhibition mitigates the severity of aortic valve fibrosis, calcification, and stenosis	rabbit	[190]
ENPP1	ARL67156	The inhibition of ENPP1 prevents the development of CAVS	rat	[191]
P2Y_2_	2-thioUTP	P2Y_2_ agonist promotes the regression of CAVS	mouse	[192]
DPP-4	Sitagliptin	DPP-4 inhibition prevents CAVS development	rabbit	[193]
Cadherin-11	SYN0012	Cad-11-blocking antibody prevents Notch1-mediated CAVS	mouse	[194]
PPARγ	Pioglitazone	PPARγ agonist attenuates the progression of aortic valve calcification	rabbit	[195]
		PPARγ agonist attenuates lipid deposition, calcification, and apoptosis in aortic valves	mouse	[196]
